# Impact of Vitamin D_3_ Functionalization on the Osteogenic Capacity of Bioinspired 3D Scaffolds Based on Ce-Doped Bioactive Glass and Spongia Agaricina

**DOI:** 10.3390/jfb16040141

**Published:** 2025-04-14

**Authors:** Ana-Maria Seciu-Grama, Sorana Elena Lazăr, Simona Petrescu, Oana Cătălina Mocioiu, Oana Crăciunescu, Irina Atkinson

**Affiliations:** 1 National Institute of Research and Development for Biological Sciences, 296, Spl. Independentei, 060031 Bucharest, Romania; ana.seciu@yahoo.com (A.-M.S.-G.); oana_craciunescu2009@yahoo.com (O.C.); 2 “Ilie Murgulescu” Institute of the Physical Chemistry of the Romanian Academy, 202, Spl. Independentei, 060021 Bucharest, Romania; slazar@icf.ro (S.E.L.); simon_pet@yahoo.com (S.P.); omocioiu@icf.ro (O.C.M.); 3 Faculty of Chemical Engineering and Biotechnologies, National University of Science and Technology Politehnica of Bucharest, 060042 Bucharest, Romania

**Keywords:** mesoporous bioactive glass, tissue scaffolds, cholecalciferol, ceria

## Abstract

Reconstruction of extensive bone defects due to age, trauma, or post-illness conditions remains challenging. Biomimetic scaffolds with osteogenic capabilities have been proposed as an alternative to the classical autograft and allograft implants. Three-dimensional scaffolds were obtained based on Ce-doped mesoporous bioactive glass (MBG) and *Spongia agaricina* (SA) as sacrificial templates functionalized with vitamin D_3_. The study aimed to investigate the effect of vitamin D_3_ functionalization on the optimal variant of a 3D scaffold doped with 3 mol% ceria, selected in our previous work based on its biological and physicochemical properties. Scanning electron microscopy (SEM) images of the non-functionalized/functionalized scaffolds revealed a porous structure with interconnected pores ranging from 100 to 350 μm. Fourier transform infrared spectroscopy (FTIR) and SEM analysis confirmed the surface functionalization. Cytotoxicity evaluation showed that all investigated scaffolds do not exhibit cytotoxicity and genotoxicity toward the Saos-2 osteosarcoma cell line. Moreover, the study demonstrated that functionalization with vitamin D_3_ enhanced osteogenic activity in dental pulp stem cells (DPSCs) by increasing calcium deposition and osteocalcin secretion, as determined by Alizarin red stain and a colorimetric ELISA kit, as a result of its synergistic action with cerium ions. The results showed that the Ce-doped MBG scaffold functionalized with vitamin D_3_ had the potential for applications in bone regeneration.

## 1. Introduction

Tissue engineering aims to develop novel materials and methods for repairing and regenerating traumatic, injured, diseased, or aging bone. While autografts remain the gold standard for bone healing, their clinical use is limited by donor site morbidity and restricted availability. Bone allografts come with high costs, potential disease transmission, and the risk of adverse immune responses [1].

Metallic alloys are primarily used as implants for large bone defects due to their mechanical strength. However, these materials can have fixation issues and, unlike natural bone, cannot self-repair or adapt to changes in physiological conditions [2]. The drawbacks of current treatments have driven scientific efforts to design biomaterials with enhanced osteogenesis, a critical area of research.

Bioactive glasses (BGs) have emerged as a promising solution to address the limitations of current bone graft materials since the discovery of 45S5-BG by Prof. Hench [3], which is considered a scaffold for bone repair [4,5]. BGs are well known for their capacity to promote bone cell growth [6,7] and form strong bonds with both hard and soft tissues [8,9]. Following implantation, these glasses undergo specific processes that result in the formation of either amorphous calcium phosphate (ACP) or crystalline hydroxyapatite (HAP) on their surface, which is crucial for their integration with the surrounding tissue [10]. Furthermore, BG are reported to release ions that trigger the expression of osteogenic genes [11,12] and stimulate angiogenesis [13,14].

However, BGs can be doped with various therapeutic elements to improve their biological properties. Rare earth elements, particularly Cerium, have been proven to promote the osteogenic differentiation of mesenchymal stem cells. Furthermore, the addition of cerium to BGs can provide anti-inflammatory, antibacterial, and antioxidant properties [15,16]. H. Mostajeran et al. [17] reported that cerium-doped BG within a polymeric matrix of an alginate-gel scaffold provided an adequate environment for the osteogenic differentiation of human bone marrow-derived mesenchymal stem cells. The paper [18] reported that the scaffold obtained from cerium-doped hydroxyapatite and cashew gum exhibits anti-inflammatory and healing properties, promising features for replacing small bone parts. Our previous study [19] produced cerium-containing MBG scaffolds (0, 1, 3 mol% ceria) using SA as a sacrificial template. Cerium-doped MBG scaffolds, especially those doped with 3 mol% ceria, demonstrated antibacterial activity, osteogenic differentiation, and non-cytotoxicity.

In addition to therapeutic ions, BG-based scaffolds can release other agents from their structure, which can stimulate tissue regeneration. Recent research has focused on surface functionalization of these scaffolds using biomolecules (e.g., growth factors (bone morphogenetic protein-2 (BMP-2), fibroblast growth factors), proteins (fibronectin derived peptides, osteonectin), sterols (liposomes, phytosterols), and hormones (calcitonin, Vitamin D_3_) [20] to create multifunctional materials that combine the properties of both the scaffolds and grafted molecules.

Vitamin D_3_ (cholecalciferol), a fat-soluble vitamin, plays a crucial role in maintaining bone structure and promoting calcium absorption, which is essential for the mineralization process, thus enhancing the overall strength and density of the regenerated bone [21]. Furthermore, vitamin D_3_ influences the expression of genes involved in osteoblast differentiation, such as Runx2 and Osterix, thereby accelerating the maturation of osteoblasts and promoting faster bone formation. It also stimulates osteoclast resorption, thereby balancing bone remodeling [22]. It can modulate the immune response at the implantation site, reducing inflammation and promoting a favorable environment for bone healing [23]. Deficiency in vitamin D_3_ is associated with various bone disorders, including osteoporosis, rickets, and impaired fracture healing [24].

Due to its crucial role in bone metabolism, integrating vitamin D_3_ into biomaterials for bone regeneration has been proposed as a method to enhance the osteogenic potential of biomaterials, such as BGs [25].

Few studies in the research literature have reported the functionalization of BG-based scaffolds with vitamin D_3_. M.E. Abad-Javier et al. reported [26] the positive effect of vitamin D_3_ on the bioactivity of the BG scaffold by accelerating the hydroxyapatite condensation on the surface. I. Negut et al. [27] demonstrated the feasibility of using thin films obtained based on BG and vitamin D_3_ to enhance osseointegration and provide corrosion protection for Ti-like implant surfaces. A. A. Gupta [28] studied a biodegradable chitosan scaffold for the sustained release of vitamin D_3_ and demonstrated its ability to deliver functional vitamin D_3_ over 3–5 days without significant cytotoxic effects. Many studies emphasize in vitro analysis, leaving a gap for in vivo data regarding biological responses, immune reactions, and bone healing, which are essential for clinical applications.

Building on the mineralization and osteogenic differentiation properties of cerium and the crucial role of vitamin D_3_ in calcium and phosphate metabolism, this research objective is to functionalize with vitamin D_3_ the optimal three-dimensional scaffold variant doped with 3 mol% ceria investigated in our earlier work and to explore the synergistic effect of cerium and vitamin D_3_ on the osteogenic differentiation capacity of bioactive scaffolds, an underexplored strategy in tissue engineering.

## 2. Materials and Methods

### 2.1. Materials

Tetraethylorthosilicate (Merck, Darmstadt, Germany), Pluronic^®^ P-123 (Sigma Aldrich, St. Louis, MO, USA), triethyl phosphate (Sigma Aldrich, St. Louis, MO, USA), calcium nitrate tetrahydrate (Carl Roth, Karlsruhe, Germany), and cerium nitrate hexahydrate (Sigma Aldrich, St. Louis, MO, USA) were used as starting materials for the synthesis of MBGs. A marine sponge (SA) (PureSponges.co.uk, Solihull, UK) was used as a sacrificial template for scaffold preparation.

### 2.2. Scaffold Preparation

The template replica method was employed for 3D scaffold preparation based on Ce-doped MBGs in the 70SiO_2_-(26-y)CaO-4P_2_O_5_-yCeO_2_ system (y denotes 3 mol% ceria) and SA, serving as template, as described in the paper [19]. Marine sponges are often used as templates because they closely resemble the architecture needed for bone scaffolds, in addition to other advantages such as biocompatibility, adjustable shape and size, and controlled degradation. The selection of 3 mol% ceria doping was based on our previous study [19].

In brief, SA was immersed in the prepared MBG solutions [19] for 15 min. Excess solution was removed by squeezing the sponge, and this process was repeated four times The green scaffold was then left at room temperature for 24 h followed by thermal treatment in two stages: I stage, heating to 700 °C, for 1 h, at a rate of 2 °C/min, and II stage up to a maximum temperature of 1200 °C at a rate of 5 °C/min.

### 2.3. Scaffold Functionalization

The scaffold surface was functionalized by immersion in a 0.03 mg/mL vitamin D_3_ [26] (Sigma-Aldrich, St. Louis, MO, USA) solution in phosphate buffer (PBS) at pH 7, subjected to continuous agitation for 20 min, at 150 rpm and 25 °C in light-protected tubes, and subsequently washed with distilled water to remove inadequately adhered vitamin D_3_. The scaffold was labeled as follows: S3Ce for the non-functionalized scaffold and S3CeD3 for the functionalized scaffold with vitamin D_3_.

### 2.4. Characterization of Functionalized/Non-Functionalized Scaffolds

Non-functionalized and functionalized Ce-doped MBG-based scaffolds were evaluated using an FEI Quanta 3D FEG microscope (FEI, Brno, Czech Republic) operating in the 5–30 kV range in high-vacuum mode coupled with energy-dispersive X-ray spectroscopy (EDS) and Fourier transform infrared spectroscopy (FTIR) conducted using a 6700 Nicolet Spectrometer (Thermo Fisher Scientific, Waltham, MA, USA).

### 2.5. Evaluation of Biological Properties

The influence of vitamin D_3_ functionalization on the biological properties of the scaffold was assessed through in vitro assays.

#### 2.5.1. In Vitro Cytocompatibility Testing

The cytocompatibility of non-functionalized and functionalized Ce-doped MBG-based scaffolds was evaluated by the extract method in the human Saos-2 osteosarcoma cell culture from the European Collection of Cell Cultures (ECACC).

Sample preparation. Before testing, the samples were washed with 75% ethanol and sterilized under UV light for 2 h. The scaffold extracts were prepared by incubating the samples in DMEM: F12 culture medium for 72 h at 37 °C. The extraction medium (25 mg/mL) was centrifuged for 5 min at 1500 rpm. The supernatant was collected, and 6 concentrations (namely 1.25, 2.5, 6.25, 12.5, 18.75, and 25 mg/mL) were tested for each sample.

Cell culture and treatment. SaOS-2 cells were cultured in T75 flasks with McCoy 5A culture medium until they achieved 80% confluence. Then, the trypsinized cells were transferred to 96-well culture plates at a density of 5 × 10^4^ cells/mL. Cells were then cultivated in McCoy 5A culture medium, maintaining a standard environment of 5% CO_2_ at 37 °C for 24 h to ensure optimal cell adhesion. After this period, the culture medium was replaced with a fresh medium containing extract concentrations ranging from 1.25 to 25 mg/mL. The plates were subsequently incubated under standard conditions for 48 and 96 h.

Cell viability assessment by MTT assay. After replacing the culture medium, the plates were incubated with 100 µL of 0.25 mg/mL MTT solution at 37 °C for 3 h. After incubation, 100 µL of isopropanol was added to the plates and the mixture was stirred for 15 min. A Sunrise microplate reader (Tecan, Grödig, Austria) was used to determine the optical density (OD) at 570 nm. A 100 µM H_2_O_2_ solution was used as a positive control. The obtained results were presented as the percentage of viable cells relative to the control sample, which consisted of cells incubated without extract and was considered to have 100% viability. The number of metabolically active cells was directly correlated with the results obtained.

Live/Dead cell viability assay. The viability of the cells was also evaluated employing the Live/Dead^®^ viability/cytotoxicity kit (Molecular Probes, Thermo Fisher Scientific, Waltham, MA, USA), as described in [29]. This method allows for dual staining of live cells with calcein (green fluorescence) and dead cells with ethidium bromide (red fluorescence). The cells were washed with PBS and treated with a solution of PBS containing 20 μM calcein-AM and 5 μM ethidium homodimer-1, following the manufacturer’s instructions after each incubation. Under standard conditions, the plates were incubated for 20 min at 37 °C. An inverted fluorescence microscope (Axio Observer D1, Carl Zeiss, Oberkochen, Germany) with a digital camera was used to capture the images. Calcein-AM staining suggests intracellular esterase activity, whereas ethidium homodimer-1 staining is linked to plasma membrane integrity.

#### 2.5.2. Genotoxicity Assessment

Genotoxicity testing was performed on the murine fibroblast cell line NCTC clone L929, cultured in MEM culture medium supplemented with 10% fetal bovine serum and 1% antibiotics, under standard conditions (37 °C temperature and 5% CO_2_ atmosphere) [30]. L929 cells were seeded at a density of 5 × 10^4^ cells/mL and allowed to adhere overnight. At 24 h after seeding, the cells were treated with the test samples at 18.75 mg/mL concentration. Following another 24 h of treatment, the cells were trypsinized and re-suspended in PBS. The positive control was incubated with 100 μM H_2_O_2_. The resulting cell suspension was mixed with a 1% LMA (low melting point agarose) solution and added onto a slide previously coated with an agarose gel. A final layer of 1% LMA agarose was added, and the prepared slides were immersed in a lysis buffer at 4 °C. The slides were deposited in an electrophoresis tank and incubated in migration buffer (1 mM EDTA, 0.3 M NaOH) to denature DNA in an alkaline environment. Migration was carried out at 0.74 V/cm for 20 min, followed by washing with Tris neutralization buffer (pH 7.5). The slides were stained with DAPI and analyzed using an Axio Observer D1 Carl Zeiss fluorescence microscope (Oberkochen, Germany) with visualization performed using a 20× objective. The median comet score, expressed as a percentage of DNA tail for each sample, was determined using Comet Score Pro v.1.5 software.

#### 2.5.3. In Vitro Osteogenic Capacity Testing

For the in vitro evaluation of osteogenic capacity, a cryopreserved culture of dental pulp stem cells (DPSCs) at passage 9 was used. DPSCs were isolated from third molars obtained from specialized clinics during the mandatory surgical or orthodontic treatment of healthy participants, with their informed consent, in accordance with the ethical regulations. The cells were preserved in liquid nitrogen until use. For the experiments, the DPSCs were cultivated in T75 flasks with α-MEM medium, 10% fetal bovine serum (FBS), 100 μg/mL streptomycin, and 100 U/mL penicillin. Until they reached 80% confluence, they were incubated in a humidified atmosphere with 5% CO_2_ and 95% air at 37 °C. These cells exhibited stem-like properties, including self-renewal, surface antigen expression, and the ability to differentiate into multiple lineages, such as osteogenic, chondrogenic, and adipogenic [31].

DPSCs were placed in a 24-well plate cell culture at a density of 5 × 10^4^ cells/mL and cultured in α-MEM with 10% fetal bovine serum (FBS) until confluence. Subsequently, the culture medium was changed with a certain osteogenic medium (Gibco, Thermo Fisher Scientific, Waltham, MA, USA) containing extract concentrations ranging from 12.5 to 18.75 mg/mL, followed by incubation under standard conditions for 14 and 21 days at 37 °C. The osteogenic marker was quantified at specific times over 21 days to evaluate the metabolic activity of the differentiated cells. A negative control group was cultured in osteogenic conditions in the absence of the extract.

Alizarin Red S staining. The formation of mineralized matrix nodules was evaluated using Alizarin Red S staining, following the protocol described in the paper, after 21 days of incubation [31]. Images were captured using an Axio Observer D1 optical microscope (Carl Zeiss, Oberkochen, Germany). Alizarin Red S-stained cells were rinsed with 2 M NaCl and incubated with 10% N-cetyl pyridinium chloride under gentle stirring for 10 min. The OD at 562 nm of the dye extract was measured using a Sunrise microplate reader (Tecan, Grödig, Austria).

Calcium quantification. The calcium colorimetric test kit (Sigma-Aldrich) was used to measure the concentration of calcium ions. In brief, 50 μL of conditioned medium collected at specific intervals was combined with 90 μL of o-cresolphthalein reagent and 60 μL of buffer to create a chromogenic complex. Afterward, the resulting mixture was incubated in the dark at room temperature for 10 min. The OD at 575 nm was measured using a Sunrise microplate reader (Tecan, Grödig, Austria). The concentration of calcium was determined based on a standard curve prepared with a 500 mM standard calcium solution.

Osteocalcin quantification. The osteocalcin concentration was measured using an ELISA kit (R&D Systems, Minneapolis, MN, USA). The concentration was ascertained using the filtrate collected following 14 and 21 days of cell treatment. OD at 450 nm was performed using a Sunrise microplate reader (Tecan, Grödig, Austria).

### 2.6. Statistical Analysis

Cell culture experiments were conducted in triplicate for each tested sample. Data are presented as mean ± standard deviation (SD) (*n* = 3). Statistical analysis was performed using a two-tailed, two-sample equal variance Student’s *t*-test (Excel v. 10). Statistically significant differences were considered at *p* < 0.05.

## 3. Results and Discussion

### 3.1. Surface Characterization of Functionalized/Non-Functionalized Ce-Containing MBG-Based Scaffolds

**Figure 1:**
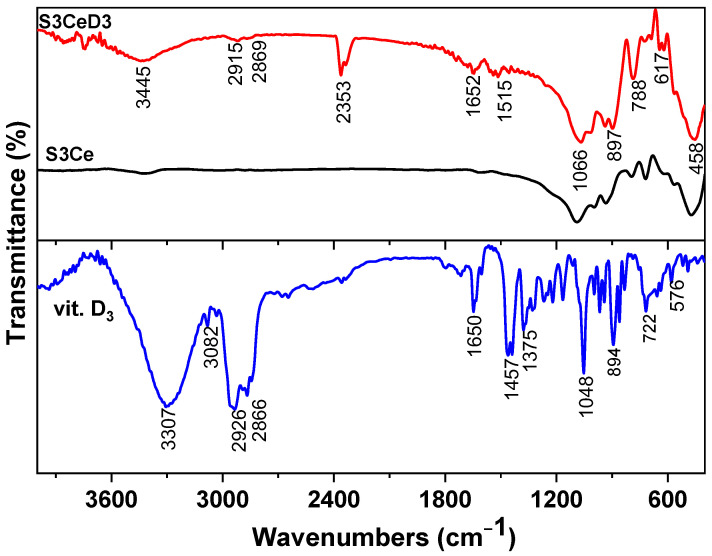
FTIR analysis of vitamin D_3_, S3Ce and S3CeD3 scaffolds.

The FTIR spectra of vitamin D_3_ along with those of the 3Ce MBG-based scaffold, before and after functionalization, are presented in Figure 1. In the vitamin D_3_ spectrum, several characteristic absorption bands were observed, indicating the presence of the functional groups: C=O stretching vibration of the aldehyde group at 1723 cm^−1^, while the C–O stretching was evident at 2352 cm^−1^. Additionally, alkyl C–H stretching vibrations were observed at 2944 cm^−1^ and 2853 cm^−1^, corresponding to the aliphatic hydrocarbon chains of vitamin D_3_. The broad band at 3420 cm^−1^ was attributed to hydrogen-bonded O–H stretching, suggesting the presence of hydroxyl groups [32].

Furthermore, the spectral region between 1645 cm^−1^ and 900 cm^−1^ exhibited multiple absorption peaks associated with C–H bending vibrations, indicative of the complex molecular structure of vitamin D_3_ [33]. In comparison, the FTIR spectra of the S3Ce scaffold before functionalization primarily displayed characteristic silicate-related vibration bands, including Si–O–Si asymmetric stretching at around 1080 cm^−1^ and Si-OH vibrations at 950 cm^−1^, as well as a phosphate group [34]. However, after vitamin D_3_ functionalization, the scaffold exhibited additional bands corresponding to the molecular fingerprint of vitamin D_3_, confirming the successful surface modification of the scaffold. The band at 2353 cm^−1^ is attributed to CO_2_ included in the vitamin D_3_ film after its exposure to air for some time, as confirmed by [35]. Characteristics bands of phosphate and silicate groups remained unchanged in the S3CeD3 scaffold, indicating that the structural integrity of the scaffold was preserved after functionalization.

**Figure 2:**
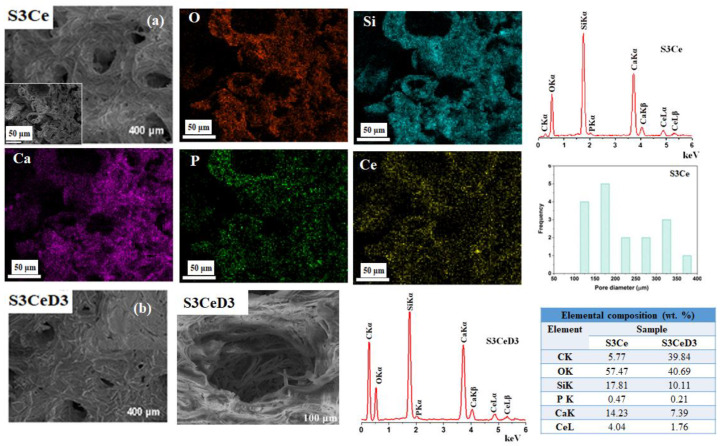
SEM image of the S3Ce scaffold, EDX mapping for O, Si, Ca, P, and Ce, EDX analysis of the S3Ce scaffold surface and pore size distribution of S3Ce scaffold (**a**); SEM image at different magnifications, EDX analysis of the S3CeD3 scaffold and elemental composition of the investigated scaffolds (**b**).

SEM images and EDX analysis further validated the surface modification (Figure 2). The SEM image of the S3Ce scaffold (Figure 2a) revealed a highly porous architecture with interconnected pores ranging from 100 to 350 μm. The scaffold demonstrated an open porosity of 87.67%, as revealed by micro-CT analysis in our previous study [19]. Porosity, interconnectivity, and pore size play a crucial role in tissue regeneration due to their direct impact on extracellular matrix formation and vascularization. Three-dimensional scaffolds with pore sizes ranging from 50 to 700 µm are commonly used in bone tissue engineering [36]. No significant changes in porosity were observed following the functionalization with vitamin D_3_.

The primary components of the S3Ce scaffold, as observed by EDX elemental mapping and analysis (Figure 2a), include calcium, phosphorus, silicon, and cerium. Notably, the EDX spectrum of the S3CeD3 scaffold (Figure 2b) showed an increased carbon signal generated by the carbon structure within the vitamin D_3_ molecule, confirming the functionalization of the scaffold surface.

### 3.2. Cytocompatibility Testing

**Figure 3:**
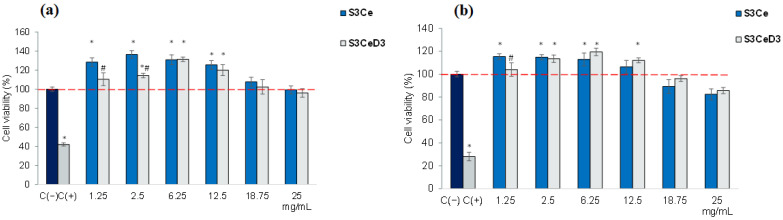
SaOS-2 osteoblast viability assessed by MTT assay in different concentrations of S3Ce and S3CeD3 scaffold extracts after 48 h (**a**) and 96 h (**b**) of cultivation. The results were obtained as the mean of three determinations ± SD and presented relative to the control culture (cells cultivated in the absence of the sample), which was considered 100% viable; * *p* < 0.05, compared to the control. # *p* < 0.05, compared to S3Ce.

The cytocompatibility of MBG-based scaffolds was analyzed using the MTT assay at concentrations ranging from 1.25 to 25 mg/mL, with cultivation periods of 48 and 96 h. The obtained results are presented in Figure 3.

The data show that treatment with the S3Ce scaffold within the concentration range of 1.25–12.5 mg/mL resulted in high cell viability of SaOS-2 osteoblasts, with values exceeding that of the untreated culture (control, 100%), at both 48 (Figure 3a) and 96 h (Figure 3b) of incubation. A slight decrease in cell viability was observed at 96 h, but the values remained above 80%, indicating good cytocompatibility at concentrations of 18.75 mg/mL and 25 mg/mL.

The incubation of SaOS-2 osteoblasts with a D_3_ functionalized scaffold resulted in high cell viability, with values exceeding that of control (100%), in the concentration range of 1.25 to 12.5 mg/mL, at 48 and 96 h (Figure 3). At higher concentrations, the cell viability remained above 80%, with values of 96.1% for 18.75 mg/mL and 85.6% for 25 mg/mL. Additionally, treatment with the S3CeD3 scaffold yielded the highest cell viability values across the concentration range of 6.25–18.75 mg/mL. Specifically, after 96 h of treatment, the viability values were 119.46% (6.25 mg/mL), 112.06% (12.5 mg/mL), and 96.1% (18.75 mg/mL) for the S3CeD3 functionalized scaffold, higher than those recorded for the non-functionalized S3Ce scaffold of 112.94% (6.25 mg/mL), 106.37% (12.5 mg/mL) and 89.24% (18.75 mg/mL). However, the values were not statistically different (*p* > 0.05).

The data obtained in the present study are consistent with other findings on vitamin D_3_-functionalized biomaterials. Bose et al. [37] reported that 3D-printed calcium phosphate scaffolds functionalized with vitamin D_3_ and quercetin showed a 1.3- and 1.6-fold increase in osteoblast cell proliferation and differentiation and reduced osteoclast activity, respectively.

**Figure 4:**
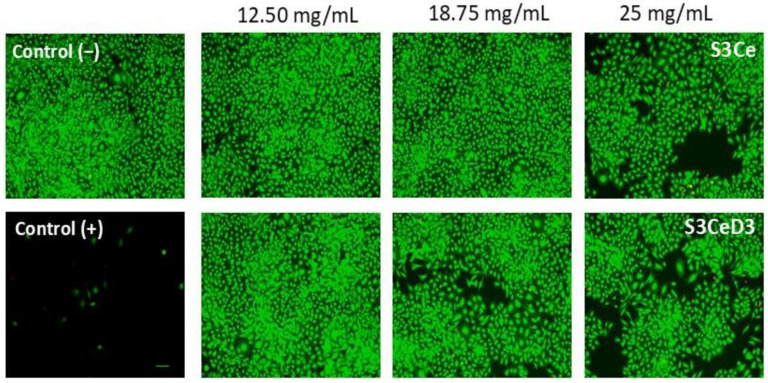
Fluorescence microscopy images indicating the SaOS-2 osteoblast viability after 96 h of cultivation in the presence of S3Ce and S3CeD3 scaffolds obtained using the Live and Dead assay.

The viability and morphology of SaOS-2 osteoblasts cultured with 3SCe and 3SceD3 scaffolds were analyzed using the Live/Dead assay after 96 h of incubation. The results presented in Figure 4 indicated that the cells treated with the tested scaffolds exhibited high viability, as evidenced by the green fluorescence of the calcein-stained live cells. The cells were uniformly distributed throughout the cultivation period and maintained a morphology similar to that of the control group. However, the cell density decreased in all analyzed scaffolds at concentrations of 18.75 mg/mL and 25 mg/mL. Despite this decrease, the cells preserved a normal morphology and uniform distribution, with only a small number of dead cells (indicated by red staining) detected.

### 3.3. Genotoxicity Testing

Considering the reported research that excessive amounts of vitamin D_3_ can lead to harmful effects on the body [38], such as alterations in cell cycle regulatory pathways [39] and increased pro-oxidation [40], the genotoxicity analysis of the scaffolds was conducted using the Comet assay (alkaline single-cell gel electrophoresis). This method is based on the property of denatured DNA fragments to migrate out of the cell under the influence of an electric field, forming the so-called “comet tails”. The larger the comet size, the greater the number of lesions in the DNA macromolecules.

**Figure 5:**
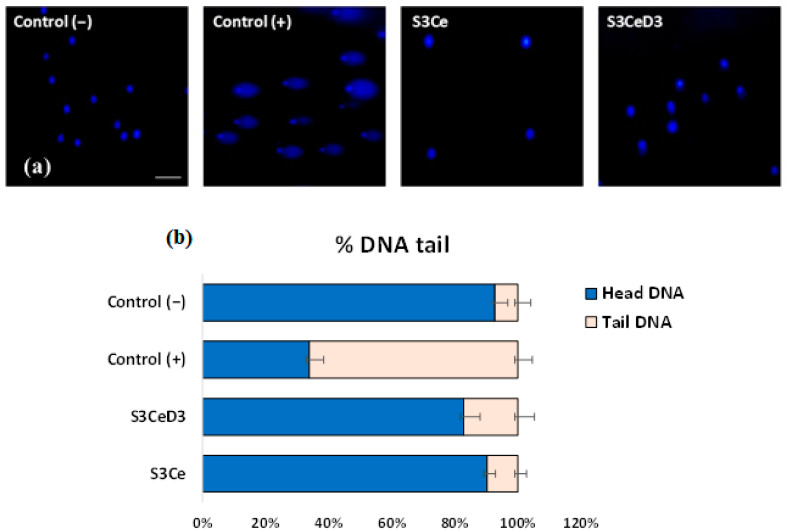
Fluorescence micrographs of DAPI-stained SaOS-2 osteoblasts cultivated in the presence of S3Ce and S3CeD3 scaffolds for 24 h (**a**) and histogram of the percentage of DNA tail in comet cells determined using Comet Score Pro v.1.5 software (**b**). The results are presented as the mean ± SD (*n* = 3). Untreated cells were used as a negative control (C (−)). Cells treated with 100 µM H_2_O_2_ were used as a positive control (C (+)). Scale bar = 100 µm.

Figure 5 presents the results of the genotoxicity analysis. Specific comet formations were observed only in the positive control group treated with 100 μM H_2_O_2_. In contrast, no comet formations were detected in the cells treated with the investigated scaffolds, and the results were comparable to those of the untreated culture control.

Using the Comet Score v. 1.5 software, the size of the comets obtained after electrophoresis in an alkaline medium was quantified. Figure 5b presents the parameter % DNA tail, representing the amount of damaged cellular DNA present in the comet’s “tail”. The results confirmed the qualitative observations and showed that the treatment with the scaffolds did not affect the treated cells, as values not significantly (*p* > 0.05) different from those of the untreated culture control were obtained.

### 3.4. Osteogenic Capacity of Functionalized Scaffolds

**Figure 6:**
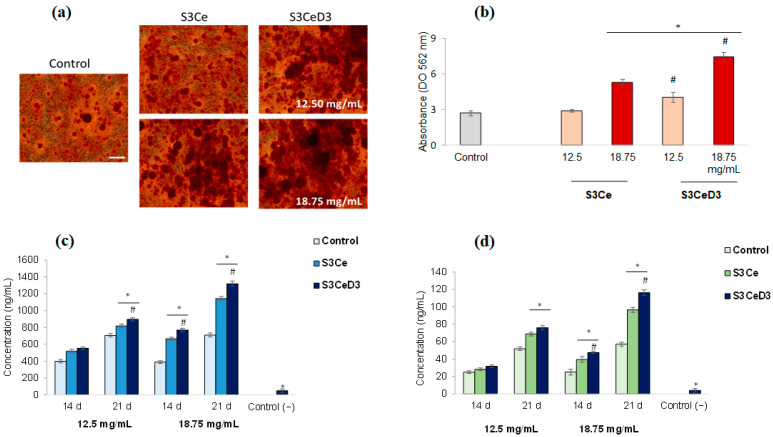
Micrographs of DPSC cultivated in osteogenic medium for 21 days in the presence of S3Ce and S3CeD3 scaffolds showing (**a**) mineralization nodules stained with Alizarin Red S and (**b**) quantitative data based on absorbance at 562 nm. * *p* < 0.05 compared to control, # *p* < 0.05 compared to S3Ce. Scale bar = 100 µm. Calcium (**c**) and osteocalcin (**d**) concentrations secreted by cells osteogenically differentiated in the presence of non-functionalized scaffolds for 14 and 21 days, estimated with a colorimetric kit and ELISA kit, respectively. Negative control: cells cultivated only in osteogenic medium. Values are expressed as the mean ± SD (*n* = 9), * *p* < 0.05 compared to control, # *p* < 0.05 compared to S3Ce.

This study on the osteogenic capacity of functionalized scaffolds aimed to provide insights into the mechanisms of biomaterial-mediated bone regeneration in a culture of osteogenic, differentiated DPSCs. Thus, the influence of the S3Ce and S3CeD3 scaffolds on the extracellular matrix mineralization of DPSCs was analyzed after 21 days of incubation. The calcium deposits in treated cultures were qualitatively and quantitatively evaluated using Alizarin Red S staining. In the untreated culture (control), Alizarin Red S staining resulted in partial red coloration of the extracellular matrix of the DPSCs, indicating the presence of small calcium or mineralized deposits. In contrast, the treated culture exhibited an intense red coloration of the extracellular matrix, particularly at 18.75 mg/mL for both non-functionalized and functionalized scaffolds (Figure 6a).

Additionally, the quantitative analysis of calcium deposit formation in the presence of functionalized MBG scaffolds indicated their increase in a dose-dependent manner, confirming the stimulation of osteogenic differentiation of stem cells. Thus, the obtained results showed that cells that osteogenically differentiated in the presence of both functionalized (S3CeD3) and non-functionalized (S3Ce) scaffolds formed significantly (*p* < 0.05) higher amounts of calcium deposits, compared to the untreated culture of stem cells, except for 12.5 mg/mL S3Ce (Figure 6b). At a concentration of 12.5 mg/mL in the presence of a S3CeD3 functionalized scaffold, an increase in calcium deposit formation 1.49 times higher than that of the untreated control was observed. At a concentration of 18.75 mg/mL, calcium deposit formation was 1.98 times higher for the S3Ce scaffold and even higher for the functionalized S3CeD3 scaffold, with values 2.77 times higher compared to the untreated control. Moreover, it was observed that at the concentration of 18.75 mg/mL, the S3CeD3 functionalized scaffold exhibited a value 1.4 times higher compared to the S3Ce scaffold. These results suggest a synergistic effect of cerium content and vitamin D_3_ functionalization, which enhanced the osteogenic capacity.

The late stage of the osteogenic differentiation of DPSCs (14–21 days) is characterized by the high expression of calcium, phosphate, and osteocalcin deposition [19]. The in vitro investigation of these markers could predict the osteogenic potential of the biomaterials after in vivo implantation. In the present study, the values obtained for the quantification of calcium ions in the culture medium of osteogenically differentiated cells in the presence of the S3CeD3 scaffold are shown in Figure 6c. After 21 days of cultivation, the S3CeD3 scaffold significantly increased calcium secretion by 1.15 times compared to the S3Ce scaffold at a concentration of 18.75 mg/mL (*p* < 0.05). This result indicates that vitamin D_3_ significantly stimulated the osteogenic capacity of Ce-doped MBG scaffolds.

Osteocalcin is an osteogenic marker synthesized by mature osteoblasts. The concentration values of osteocalcin secretion by cells osteogenically differentiated in the presence of non-functionalized and functionalized scaffolds are presented in Figure 6d. The results show a similar concentration variation to that observed for calcium ion secretion at 14 and 21 days of cultivation. After 21 days, osteocalcin secretion was 1.2 times higher at a concentration of 18.75 mg/mL when using the functionalized S3CeD3 scaffold compared to the non-functionalized S3Ce scaffold.

The obtained results, an increase in calcium ion secretion by 1.15 times and osteocalcin levels by 1.2 times by the S3CeD3 functionalized scaffold, compared to the S3Ce scaffold after 21 days of incubation, align with other work showing that vitamin D_3_-functionalized scaffolds enhance osteocalcin expression and mineralization via integrin-mediated signaling and MAPK/ERK pathway activation [41]. The osteogenic properties of this latter scaffold were positively modulated by the polyurethane foam templating, which provided a grain-like microtopography, upregulating the expression of osteogenic genes through the MAPK/ERK signaling pathway activated by the adsorption of fibronectin and the expression of integrin α5β1 and focal adhesion kinase (p-FAK) [41]. Another study has revealed an increase in mineral deposition, as observed using Alizarin red staining, in human adipose-derived stem cells incubated with a polycaprolactone/gelatin scaffold on day 21 [42].

Osteogenesis is a complex process involving several transcription factors that govern the differentiation of mesenchymal cells into osteoblasts. The key transcription factor responsible for initiating the osteodensification of mesenchymal cells is runt-related transcription factor 2 (Runx2). Once these cells differentiate into pre-osteoblasts, Runx2, osterix, and β-catenin direct their development into immature osteoblasts that produce specific proteins [43]. The Runx2 expression must be decreased for bone maturation to continue because it inhibits osteoblast maturation by maintaining its immature state. Osteocalcin is the most abundant non-collagenous protein found in bones. It is considered a suitable marker for osteogenic maturation, and it is regarded as a late indicator of osteodifferentiation. Mature osteoblasts express high levels of osteocalcin when they are finally embedded in the bone matrix and transform into osteocytes [44].

The functionalization of the scaffold creates a biomimetic microenvironment that mimics the native extracellular matrix (ECM) of bone, fostering cell-matrix interactions crucial for osteogenesis.

The contribution of Ce doping to osteogenesis lies in its antioxidant properties, resulting from the presence of Ce^3+^/Ce^4+^ at the scaffold surface, which can catalyze the dismutation reaction of hydrogen peroxide, thereby mitigating reactive oxygen species (ROS) and creating a favorable environment for osteoblast activity. Our previous study determined a Ce^4+^/Ce^3+^ ratio of ~0.5 for the S3Ce scaffold using UV-Vis spectroscopy [19]. Jinhua Li et al. [45] reported a close relationship between the Ce^4+^/Ce^3+^ ratio and bone formation, indicating that a higher Ce^4+^/Ce^3+^ ratio promotes better osseointegration. Ce^3+^ scavenges ROS, protecting cells from apoptosis and enhancing their responsiveness to osteogenic stimuli like vitamin D_3_.

Vitamin D_3_ enhances osteogenic differentiation through multiple mechanisms, including the activation of key osteogenic markers such as RUNX2, osteocalcin, and alkaline phosphatase, regulation of calcium–phosphate homeostasis, stimulation of osteoblast differentiation, and modulation of signaling pathways, such as the β-catenin signaling pathway [46].

This synergistic interplay between Ce and vitamin D_3_ may explain why the S3CeD3 scaffold exhibited superior performance: Ce scavenged reactive oxygen species (ROS), while vitamin D_3_ directly stimulated osteogenic differentiation. Combining the antioxidant benefits of Ce with the osteogenic signaling of vitamin D_3_ represents a promising approach. While our findings highlighted their potential to enhance the osteogenic differentiation of the scaffold, further studies are necessary to elucidate the mechanisms involved in osteogenesis and ultimately translate these findings into clinical applications.

## 4. Conclusions

Three-dimensional scaffolds were obtained by doping MBGs with 3 mol% ceria and SA and then functionalized with vitamin D_3_.

SEM and FTIR investigations confirmed the successful functionalization of the S3CeD3 scaffold. In vitro testing confirmed the cytocompatibility of the functionalized scaffolds, promoting the proliferation of SaOS-2 osteoblasts in a higher proportion than the non-functionalized variants.

The cytotoxicity and genotoxicity evaluation confirmed the safe use of the investigated scaffolds and their potential for biomedical applications. An increase in calcium concentration by 1.15 times and osteocalcin levels by 1.2 times, as well as the formation of mineralization nodules, were observed in experiments on the osteogenic differentiation of DPSCs using the vitamin D_3_-functionalized scaffold compared to a non-functionalized one. These results confirmed the synergistic effect of cerium doping and vitamin D_3_ functionalization on MBG scaffolds, improving their osteogenic potential.

Future studies, particularly those utilizing in vivo animal models, should evaluate osteogenesis differentiation and confirm its potential clinical applications.

## Data Availability

The data presented in this study are contained within the article.
